# A lifetime of pulmonary gas exchange

**DOI:** 10.14814/phy2.13903

**Published:** 2018-10-22

**Authors:** John B. West

**Affiliations:** ^1^ Department of Medicine University of California, San Diego La Jolla California

**Keywords:** Carbon dioxide retention, hypoxemia, MIGET, ventilation‐perfusion ratio

## Abstract

Pulmonary gas exchange is the primary function of the lung, and during my lifetime, its measurement has passed through many stages. When I was born, many physiologists still believed that the lung secreted oxygen. When I was a medical student, the only way we had to recognize defective gas exchange was whether the patient was cyanosed. The advent of the oximeter soon showed that this sign could be very misleading. A breakthrough was the introduction of blood gas electrodes that could measure the PO_2_, PCO_2_, and pH of a small sample of arterial blood. It was soon recognized that the commonest cause of hypoxemia was ventilation‐perfusion inequality, and that this could also be responsible for CO_2_ retention. In the early days, the understanding of the mechanisms of pulmonary gas exchange relied on graphical analysis because the oxygen and carbon dioxide dissociation curves are nonlinear and interdependent which precluded algebraic methods. However, with the introduction of digital computing, problems that had hitherto been impossible to tackle became amenable to study. A key advance was the development of the Multiple Inert Gas Elimination Technique. Now, noninvasive methods for measuring gas exchange show promise, and the whole subject continues to develop.

I have spent much of my life thinking about pulmonary gas exchange. Looking back, I see enormous changes in the topic, and looking forward I anticipate fascinating new developments. During my lifetime, knowledge has progressed from the incorrect belief that the lung secreted oxygen, to sophisticated computer analyses of gas exchange. Many students (and perhaps their mentors) do not appreciate the speed of change in respiratory physiology, and gas exchange is an engaging example.

## Very Early Days

I embarked on pulmonary gas exchange at the age of about 30 sec, although naturally I do not recall this. It is just as well that I do not remember, because being born must be the most cataclysmic event in most peoples’ lives. What with gas exchange changing from placental to pulmonary within a few seconds, the enormous respiratory efforts required to expand the poorly inflated lung, the dramatic decrease in pulmonary vascular resistance, the closure of the ductus arteriosus, and the traditional whacks on the behind, it is a mercy that all this is forgotten.

## Oxygen Secretion by the Lung

My next few years were uneventful, but when I was 7 years old, a landmark in the history of pulmonary gas exchange occurred. This was the publication of the second edition of J. S. Haldane's book titled Respiration (Haldane and Priestley 1935). The book included an entire chapter devoted to the subject of oxygen secretion by the lung and it makes excellent reading even today. Haldane was one of the most eminent physiologists of the early 1900s, and he made important contributions to many areas including the control of ventilation, the measurement of respiratory gases in both air and blood, decompression sickness, and the detection of noxious gases in mines. However, he was something of a vitalist. Like many scientists before him, he could not believe that the extraordinary properties of living creatures could be fully explained by the simple laws of chemistry and physics.

Haldane's belief in oxygen secretion by the lung was partly based on measurements of the oxygen concentration in fish swim bladders. This is the organ that controls the buoyancy of the fish, and it was known that the partial pressure of oxygen in the bladder often greatly exceeded that in the water around the fish. Haldane concluded that the explanation was that the bladder secreted oxygen. Now the swim bladder develops from the primitive gut just as does the lung, and Haldane argued that if the swim bladder could secrete oxygen, why not the lung. He pointed out that this could be of great assistance to maintaining the PO_2_ in the body, especially at high altitude. We now know that the high partial pressures of oxygen in the fish swim bladder are caused by a counter‐current gas exchange mechanism.

During the Anglo‐American high altitude expedition to Pikes Peak, Colorado, in 1911 when Haldane was one of the leaders, he reported that he had found evidence for substantial oxygen secretion by the lung as evidenced by the fact that the arterial PO_2_ was as much as 35 mm Hg higher than the alveolar value, particularly in subjects acclimatized to high altitude. This was not the case but even today it is not entirely clear how this error occurred, although the method of measuring the arterial PO_2_ was very indirect. The theory of oxygen secretion was vigorously attacked by Krogh (1910) and others, and we now believe that oxygen always crosses the blood gas barrier by passive diffusion.

## Cyanosis

Now jump to the time when I was a medical student. Amazingly, the only way to determine whether a patient had defective pulmonary gas exchange was to look for signs of cyanosis. Blood gas measurements were far into the future. I can remember tutorials where we were shown how to pull down the skin, just below the eye, and look for a blue tinge in the conjunctiva. The color of the tongue was also an important sign. It is true that arterial puncture to obtain a sample of arterial blood had been demonstrated long before. For example, Stadie (1919) used this to measure the arterial oxygen saturation in patients with pneumonia, and compare these with estimates of cyanosis. In addition, methods for measuring the oxygen saturation of the blog had been developed, for example by Lundsgaard and Van Slyke (1923). They found that approximately 5 g of reduced hemoglobin needed to be present in 100 mL of capillary blood for visible cyanosis to be present. However, those procedures were far too complicated to be used in ordinary clinical practice.

The unreliability of assessing the gas exchange function of the lung from cyanosis was emphasized in the late 1940s by Comroe and Botelho (1947). In an extensive study of 127 observers, they found that the majority were unable to detect the presence of definite cyanosis until the arterial oxygen saturation fell to approximately 80%, and that 25% of the observers did not report definite cyanosis even at arterial saturation levels of 71 to 75%. Note that an oxygen saturation of 80% meant that the arterial PO_2_ would only be about 45 mm Hg for a normal oxygen dissociation curve, so severe hypoxemia was present. Nevertheless, the physical sign of cyanosis continued to be emphasized until much later, primarily because no other practical methods of measuring oxygen levels in the blood were available. This was certainly the case when I was a newly minted doctor in 1952.

## Oximetry and Expired Gas Analysis

A major advance in the clinical assessment of abnormal pulmonary gas exchange was the introduction of the ear oximeter. This was first described by Millikan (1942) in the US and Kramer in Germany. Millikan's device was improved by Earl Wood at the Mayo Clinic who added a pneumatic cuff to obtain a bloodless zero. Two wavelengths of light in the red and infrared regions were used. In fact, it was the advent of this oximeter that allowed Comroe and Botelho to carry out the study on the recognition of cyanosis that was referred to in the previous paragraph. The original instrument was delicate because it included a small mirror supported by a fine quartz fiber that was deflected as the light transmitted through the ear changed.

**Figure 1:**
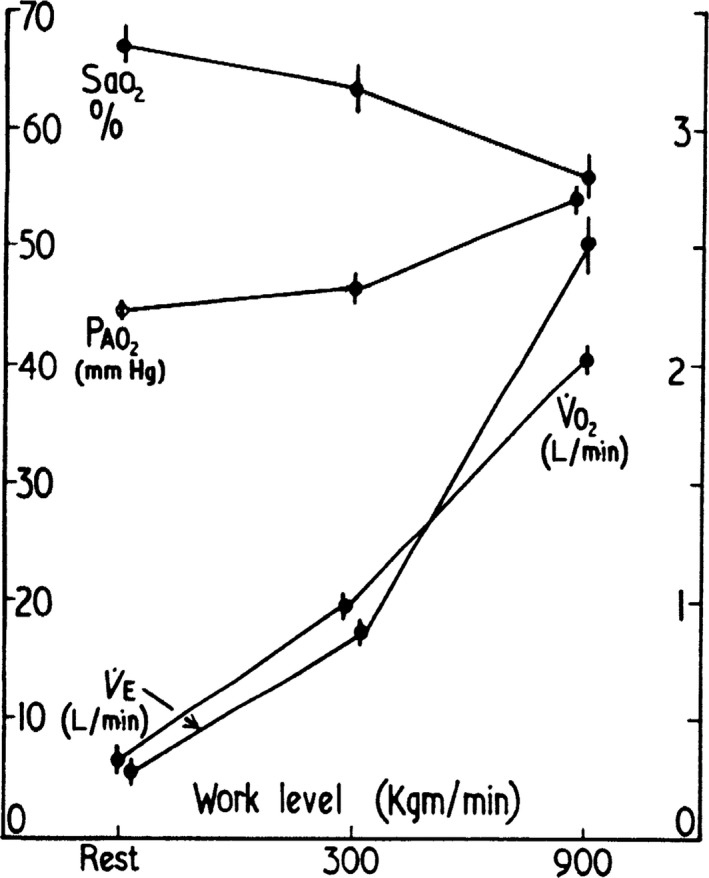
Fall in arterial oxygen saturation with increasing work level in spite of a rise in the alveolar PO_2_. These measurements were made at an altitude of 5800 m. This innovative study was done with a very early oximeter. From West et al. (1962).

I used this instrument during the Himalayan Silver Hut Expedition of 1960 to measure the arterial oxygen saturation in us at a very high altitude. The altitude of the hut in which we lived for several months was 5800 m (19,000 ft) and the barometric pressure was only 380 mm Hg, which is half the sea level value. We exercised to our maximal level and the results are shown in Figure 1 (West et al. 1962). At rest, the oxygen saturation was only 67% at this very high altitude. However, when the work level was increased to an oxygen uptake of about 1 L per minute, the saturation fell further to 63%, and at an oxygen consumption of about 2 L per minute, the oxygen saturation was only 56%. For blood with a normal P_50_, this corresponds to an arterial PO_2_ of only about 27 mm Hg, although it is likely that these subjects had an increased oxygen affinity of the hemoglobin because the very high ventilations caused a respiratory alkalosis.

Figure 1 also shows that these large falls in oxygen saturation occurred in the face of an increasing alveolar PO_2_ that rose from about 45 to 53 mm Hg as the work level was raised. The explanation for the dramatic fall in oxygen saturation under these conditions was diffusion limitation across the blood gas barrier. It is usual to explain the diffusion limitation at low alveolar PO_2_ values to the reduced head of pressure of oxygen, but a more accurate analysis shows that the main reason is that the slope of the oxygen dissociation curve is very steep. It was possible to relate the observed results to theoretical predictions based on reasonable values for the diffusing capacity of the lung. In re‐reading the article after 50 years, it is a little alarming to see that one of the saturation measurements in myself was 33% and this was measured in duplicate.

These are particularly interesting measurements because virtually the only situation where substantial decreases in arterial oxygen saturation are seen in normal subjects during heavy exercise is at very high altitude. It is remarkable that the results were obtained under such difficult conditions with such a primitive instrument. Long before, Barcroft (1925) had predicted that desaturation would be seen under these conditions based on measurements that he made in Peru at an altitude of 4300 m.

These studies from the 1960 Silver Hut expedition were so successful that we subsequently wondered whether it might be possible to obtain physiological measurements on normal subjects at even higher altitudes, possibly on the summit of Mount Everest, the highest point in the world. That opportunity occurred in 1981 during the American Medical Research Expedition to Everest, which I led. Three American climbers reached the summit and several crucial physiological measurements were made. Some of the most interesting data were obtained by collecting samples of alveolar gas using a specially designed piece of equipment that trapped the last expired gas from a maximal expiration. The samples were subsequently brought back to the University of California, San Diego for analysis, and some of the results are shown in Table 1 (West et al., 1983). Note that the barometric pressure (which the expedition measured for the first time), was only 253 mm Hg, that is one‐third of the normal sea level value. The inspired PO_2_ was therefore 43 mm Hg, and the alveolar PO_2_ from the gas samples was only 35 mm Hg. However, the calculated arterial PO_2_ was lower because of diffusion limitation across the blood gas barrier, the value being about 28 mm Hg. The arterial PCO_2_ based on the alveolar value was extraordinarily low at about 7.5 mm Hg. We were able to derive the arterial pH based on this PCO_2_ because the base excess was measured on two of the climbers. The pH exceeded 7.7 (Table 1). It was certainly one of the highlights of my interest in pulmonary gas exchange to be able to report these values from the highest point in the world.

**Table 1:** Pulmonary gas exchange on the summit of Mount Everest. From West et al. (1983)

Altitude meters	Barometric pressure mm Hg	Inspired PO_2_ mm Hg	Alveolar PO_2_ mm Hg	Arterial PO_2_ mm Hg	Arterial PCO_2_ mm Hg	Arterial pH
8848 (summit)	253	43	35	28	7.5	>7.7
Sea level	760	149	100	95	40	7.40

Returning to oximetry, this has made enormous strides in the last 30 years, and the introduction of the modern pulse oximeter has played a key role in assessing pulmonary gas exchange in a large variety of settings including emergency medicine and anesthesia to name just two (Severinghaus and Honda 1987). The improvement exploited the fact that looking at the variation in the optical density of tissues at two wavelengths during the normal pulsation caused by the heart beat can give the oxygen saturation. These pulsations obviate the need for a bloodless optical path that was necessary in the early oximeters. The small instrument is typically applied to a finger but in infants the foot can be used. The accuracy of the pulse oximeter that is now used by the thousands has been extensively tested, and generally the reading is within about 2% of the actual arterial oxygen saturation.

Although the oximeter was an important advance in understanding gas exchange, our knowledge was still dismal when I was a medical resident. The rudimentary state of clinical physiology, particularly that of respiration and biochemistry in the early 1950s, was highlighted during the Copenhagen poliomyelitis epidemic of 1952. This took place when I had just graduated from medical school although I was not aware of this disaster at the time. The tragedy was that over the course of a few weeks, more than 300 patients developed respiratory paralysis, that is they were not able to breathe, and the ventilator facilities at the hospital were completely inadequate. As a consequence, 200 medical students were prevailed upon to provide ventilation by hand for 24 h a day, using a rubber bag attached to a tracheostomy tube. This unprecedented crisis makes dramatic reading (West 2005).

The interpretation of the blood chemistry was challenging and was confusing at first. The only measurement available at the time was the carbon dioxide concentration in the blood. Measurements of this showed very high values and these were initially attributed to a mysterious “alkalosis”. But when a crude pH meter was made available, it was clear that the patients had a striking acidosis, and it was recognized that there was a high PCO_2_ caused by hypoventilation. This desperate situation heralded the beginnings of modern acid‐base clinical physiology.

## Blood Gas Analysis

Oximetry was useful in giving the oxygen saturation of arterial blood, but this was a very inexact way of determining the arterial PO_2_. The reason is the very nonlinear shape of the oxygen dissociation curve. For example, the arterial PO_2_ can fall from 100 to 60 mm Hg, that is a change of 40%, while the oxygen saturation only changes from 97 to 90%, that is a reduction of 7%. Therefore, a great deal of attention was devoted to developing methods to measure the PO_2_ in the blood.

Initially, this was done by using an aerotonometer in which a small bubble of air was equilibrated with blood and then analyzed. Early attempts to do this were made in the 1870s, but later Roughton and Scholander (1943) developed a simpler syringe method for gas analysis. In this, a bubble of gas was equilibrated with a sample of blood in a small syringe, and the volume of gas was measured in a fine capillary tube. This method was improved by Riley and Cournand (1949), and their subsequent understanding of the factors determining the arterial PO_2_ had a large influence as briefly discussed below.

However, the bubble technique was slow and technically demanding, and the measurement of arterial PO_2_ in blood was revolutionized by the introduction of the polarographic oxygen electrode. The principle of this is that a small potential difference of 0.6 V is maintained between two electrode tips in the blood, and the resulting current is proportional to the PO_2_. Early forms of this device used a mercury dropping electrode, but this was replaced by a platinum electrode by Clark et al. (1953). Originally, the electrode was covered with cellophane, but this was prone to errors because of oxygen depletion near the electrode. This problem was then avoided by using a very small electrode tip.

Soon after the development of the oxygen electrode, Severinghaus and Bradley (1958) described an electrode for measuring the PCO_2_ in blood. They arranged to have the CO_2_ diffuse from the blood through a thin membrane into a small compartment housing a pH electrode. Soon the oxygen and carbon dioxide electrodes were mounted in a single commercially available package that was temperature controlled. The result was the ability to make measurements of arterial PO_2_, PCO_2_, and pH of a small sample of blood. This has revolutionized our understanding of pulmonary gas exchange.

## Ventilation‐Perfusion Relationships

When it was possible to measure the PO_2_, PCO_2_ and pH in arterial blood, physiologists were stimulated to analyze the factors responsible for the gas exchange abnormalities. Great strides in understanding defective gas exchange were made during World War II, and the story is interesting. At the University of Rochester NY, Wallace Fenn had assembled a group of three physiologists with a variety of skills. Fenn's main interest was muscle energetics, and he is known for the “Fenn effect” that describes the heat induced by muscle contraction. Hermann Rahn had worked earlier on the pituitary gland of birds, and more recently on the reproductive behavior of rattlesnakes. Arthur Otis had studied the effects of drugs on the oyster heart, and then had worked on the development of grasshopper eggs. None of these three investigators had a particular interest in respiratory physiology, and it seemed a very unlikely trio to make advances in this area. However, with the attack on Pearl Harbor in December 1941 everything changed, and because of the exigencies of war, the group was directed to study aviation physiology. In the event, they laid many of the foundations of both pulmonary gas exchange and mechanics.

The initial projects had to do with pressure breathing because it was thought that raising the pressure of the inspired gas would give an aviator an advantage in flying at a high altitude. This prompted an analysis of the composition of alveolar gas under a variety of conditions. A major advance was the introduction of the oxygen‐carbon dioxide diagram with PO_2_ on the horizontal axis and PCO_2_ on the vertical axis. This greatly clarified the understanding of alveolar gas composition under different conditions. For example, it was found that the respiratory exchange ratio of a gas could be easily determined from a series of straight lines radiating from the point representing the composition of inspired gas.

**Figure 2:**
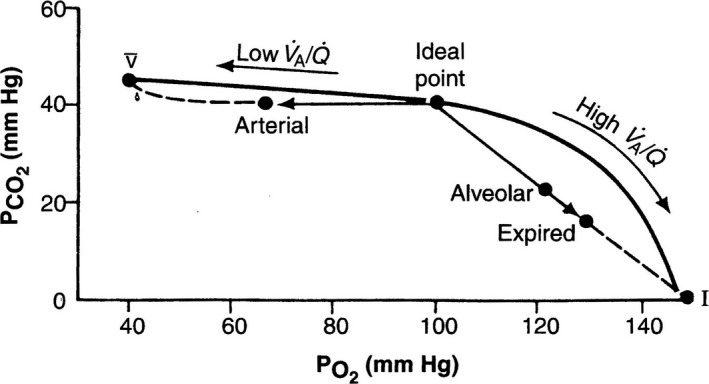
The classical O_2_ – CO_2_ diagram showing the R lines for gas and blood, and the ventilation‐perfusion line that joins the mixed venous and inspired gas points. Modified from Rahn and Fenn (1955).

It was soon discovered that it was possible to add the blood partial pressures to the diagram, and this clarified the gas exchange between alveolar gas and pulmonary capillary blood. A major advance was recognizing that it was possible for the O_2_‐CO_2_ diagram to depict the effects of changing the ratio of ventilation to blood flow on gas exchange. This analysis culminated in drawing a ventilation‐perfusion ratio line from the mixed venous blood point to the inspired gas point. This line is remarkably powerful because it depicts all possible partial pressures of gas and blood in lung units for a lung supplied with inspired air, and a particular composition of mixed venous blood (Fig. 2).

While these important advances were being made, another group including Richard Riley and Andre Cournand at Bellevue Hospital in New York City were also studying defective gas exchange. Riley started by considering the gases in blood partly because, as indicated earlier, he was responsible for developing the method for determining the PO_2_ and PCO_2_ in blood by adding a small bubble of gas and then analyzing its composition. As a result, Riley and coworkers developed a special interest in the transfer of oxygen from alveolar gas to pulmonary capillary blood. They recognized, as had Fenn's group, that in the diseased lung, the alveolar gas composition varied between different lung units depending on the ventilation‐perfusion ratio. They introduced the notion of what they called “ideal” alveolar gas. This is the composition that the alveolar gas would have if it was uniform in composition, and had the same respiratory exchange ratio (CO_2_ output divided by O_2_ uptake) as the actual lung.

Their method for determining the composition of ideal alveolar gas was to use the measured arterial PCO_2_, and the alveolar gas equation that relates the alveolar PO_2_ to the inspired PO_2_, the arterial PCO_2_, and the R value. The justification for using the arterial PCO_2_ for the alveolar value was that the blood R line is almost horizontal, and therefore the arterial PCO_2_ must be close to the ideal value (Fig. 2). This somewhat abstruse derivation has turned out to be clinically useful. In a situation where the efficiency of pulmonary gas exchange needs to be assessed, a useful metric is the alveolar‐arterial PO_2_ difference, where the alveolar value is given by the ideal gas, and the arterial value is directly measured from blood.

There is an anecdote about Riley's novel conception of ideal alveolar gas. He had moved from Bellevue Hospital to the Johns Hopkins Medical School in Baltimore, and developed pulmonary tuberculosis, a common event among physicians at the time. The treatment was bed rest, and he had plenty of time to muse about the mysteries of pulmonary gas exchange. As he wrote in a personal account “never was enforced confinement given more profitable psychotherapy” (Riley 1980).

## The Advent of Digital Computing

The analysis of pulmonary gas exchange as described by the groups of Fenn et al. and Riley and Cournand was not amenable to a formal algebraic approach because of the nature of the oxygen and carbon dioxide dissociation curves. These are not only nonlinear, but they are also interdependent. For example, the oxygen dissociation curve is very nonlinear, and its position is altered by the prevailing PCO_2_, and other factors. These complications are the reason for the graphical approaches adopted by both groups. This area of research culminated in 1955 when Rahn and Fenn published a classical booklet on the analysis of pulmonary gas exchange and the O_2_‐CO_2_ diagram (Rahn and Fenn 1955). I devoured this, and fell in love with the topic. As a result, I spent a year in Rahn's department in the University of Buffalo, NY.

However, a sea change occurred in the 1960s when the introduction of digital computing showed that it was possible to solve problems in pulmonary gas exchange that had been impossibly complicated before. The leaders in this revolution included Kelman (1967) in the UK, and Olszowka and Farhi (1969) in the US. Shortly after these papers appeared, I was fortunate to spend a year in a NASA center, the Ames Research Center. This had excellent computing facilities because it had a strong engineering program with some of the largest wind tunnels in the country. The main reason why I was in this center is that the Apollo program to put a man on the moon and bring him back alive was in full swing. I had worked extensively on the effects of gravity on the lung. For example, we had discovered the very uneven topographical distribution of pulmonary blood flow that is caused by gravity, and I thought it would be fascinating to see what happens in the weightlessness of space. However, I was also able to take full advantage of the sophisticated computing facilities.

I spent much of the year developing a computer model of a lung with ventilation‐perfusion inequality. It was soon clear that some of the questions that previously had been impossibly difficult to solve would become amenable to analysis. To take one example, I was very surprised to find that when a lognormal distribution of ventilation‐perfusion ratios was imposed on a normal lung, the reduction in carbon dioxide transfer was almost as great as that for oxygen transfer. This had important implications for understanding the mechanism of CO_2_ retention in the severe chronic obstructive pulmonary disease (West 1971). I wrote up the results of these studies in some detail (West 1969) and the project gave me a lot of satisfaction.

## The Multiple Inert Gas Elimination Technique (MIGET)

One of the most important products of the application of digital computing to the diseased lung models has been the Multiple Inert Gas Elimination Technique developed by my colleague Peter Wagner. I like to think that this was initiated in part by my studies on computer models of the lung referred to above, but MIGET, as it is called, goes far beyond anything that I have done. The details are too complicated to describe here but basically, instead of relying on the gas exchange of the naturally occurring gases, oxygen and carbon dioxide, MIGET involves infusing a solution of a cocktail of six gases of very different solubilities into a peripheral vein. The concentrations of these gases in alveolar gas and arterial blood are then measured, and the computer then finds a distribution of ventilation‐perfusion ratios that is compatible with the data. The end result is a far more sophisticated insight into the effects of ventilation‐perfusion inequality than has previously been available.

**Figure 3:**
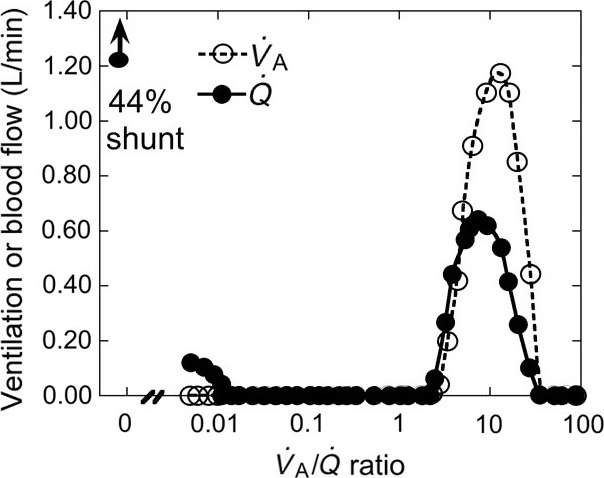
Example of the potential of MIGET. The plot shows the ventilation‐perfusion relationships in a patient with ARDS. There is a large shunt and a region of the lung that has a nearly normal pattern of ${\dot{V}}_{A}/\dot{Q}$. ratios. However the mean ${\dot{V}}_{A}/\dot{Q}$ is very high implying that the blood flow has been greatly decreased. The probable reason is the positive pressure ventilation. From Hopkins and Wagner (2017).

The mathematical techniques involved in the procedure are very sophisticated, and were developed in conjunction with John Evans who was a professor of mathematics at UCSD. An example of the type of output that can be obtained with MIGET is shown in Figure 3. This shows a very abnormal distribution in a patient with ARDS. There is a large shunt of nearly half the total blood flow. However, the most interesting feature is the pattern in the nonshunt region. The diversion of blood to the rest of the lung would be expected to double the mean of the blood flow there. But as can be seen, the mean ventilation‐perfusion ratio is about 10 times normal. This must mean that something else is going on to reduce the blood flow. A likely cause is the positive pressure ventilation, which is presumably reducing the cardiac output. This distribution is giving detailed information about the gas exchange abnormality that could not be obtained in any other way. Wagner and a coworker have written a detailed account of MIGET in a book (Hopkins and Wagner 2017). This is not bedtime reading but shows the great level of sophistication that MIGET has opened up.

## Noninvasive Measurement of Impaired Pulmonary Gas Exchange

All the presently available techniques for assessing the efficiency of pulmonary gas exchange include taking a sample of arterial blood. This has some disadvantages. It can be uncomfortable for the patient, it requires an experienced technician, there are occasional complications, and the procedure is time‐consuming and expensive. Now, (90 years after the first breath that began this review) we are working on a noninvasive method of assessing pulmonary gas exchange in patients with lung disease. This exploits advances in technology that enable the continuous measurement of inspired and expired PO_2_ and PCO_2_ using miniaturized sensors that can be taken to the bedside. A pulse oximeter is used to calculate the arterial PO_2_ using the oxygen dissociation curve while taking account of the effects of changes in PCO_2_ using the alveolar value. The difference between the end‐tidal PO_2_ and the calculated arterial PO_2_ is known as the Oxygen Deficit.

Measurements show that this oxygen deficit is very small in young normal subjects, and it increases slightly with age as would be expected. Studies in a series of patients with various types of lung disease show that the oxygen deficit increases greatly and it is a very sensitive index of abnormal gas exchange. The test is very quick and simple to do. The patient breathes through a mouthpiece for 2 or 3 min until a state of gas exchange has been established, and the device then immediately displays the oxygen deficit. It is too early to say whether this new development will be useful clinically, but the prospects are encouraging (West et al. 2017).

## Summary

The measurement of pulmonary gas exchange, which is the fundamental function of the lung, has passed through many stages during my lifetime. When I was born, some physiologists believed erroneously that oxygen was actively secreted by the lung. When I was a medical student, the only way we had for detecting defective gas exchange was whether the patient was cyanosed. However with the development of the oximeter, it was soon shown that cyanosis was a very poor metric. A breakthrough was the development of electrodes that could measure PO_2_, PCO_2_, and pH in a sample of arterial blood. A consequence of this was recognizing the importance of ventilation‐perfusion inequality, and we now know that this is responsible for most of the hypoxemia and CO_2_ retention in lung disease. The advent of digital computing has made a dramatic change in our understanding of pulmonary gas exchange, and one of the results has been the Multiple Inert Gas Elimination Technique that has clarified many areas that were previously obscure. Finally, it seems possible that a noninvasive measurement of pulmonary gas exchange is in the wings. The whole area has been enormously exciting, and it was a privilege to be active during these developments.

## Conflict of Interest

None declared.
